# The metabolic impact of extracellular nitrite on aerobic metabolism of *Paracoccus denitrificans*

**DOI:** 10.1016/j.watres.2017.02.011

**Published:** 2017-04-15

**Authors:** K.R. Hartop, M.J. Sullivan, G. Giannopoulos, A.J. Gates, P.L. Bond, Z. Yuan, T.A. Clarke, G. Rowley, D.J. Richardson

**Affiliations:** a School of Biological Sciences, University of East Anglia, Norwich Research Park, Norwich, NR4 7TJ, UK; b Advanced Water Management Centre (AWMC), University of Queensland, St Lucia, Brisbane, QLD, 4072, Australia

**Keywords:** Nitrite, Free nitrous acid, Denitrification, *Paracoccus denitrificans*, Nitrosative stress, Reactive nitrogen species, Flavohemoglobin, Nitric oxide

## Abstract

Nitrite, in equilibrium with free nitrous acid (FNA), can inhibit both aerobic and anaerobic growth of microbial communities through bactericidal activities that have considerable potential for control of microbial growth in a range of water systems. There has been much focus on the effect of nitrite/FNA on anaerobic metabolism and so, to enhance understanding of the metabolic impact of nitrite/FNA on aerobic metabolism, a study was undertaken with a model denitrifying bacterium *Paracoccus denitrificans* PD1222. Extracellular nitrite inhibits aerobic growth of *P. denitrificans* in a pH-dependent manner that is likely to be a result of both nitrite and free nitrous acid (p*K_a_* = 3.25) and subsequent reactive nitrogen oxides generated from the intracellular passage of FNA into *P. denitrificans*. Increased expression of a gene encoding a flavohemoglobin protein (Fhp) (Pden_1689) was observed in response to extracellular nitrite. Construction and analysis of a deletion mutant established Fhp to be involved in endowing nitrite/FNA resistance at high extracellular nitrite concentrations. Global transcriptional analysis confirmed nitrite-dependent expression of *fhp* and indicated that *P. denitrificans* expressed a number of stress response systems associated with protein, DNA and lipid repair. It is therefore suggested that nitrite causes a pH-dependent stress response that is due to the production of associated reactive nitrogen species, such as nitric oxide from the internalisation of FNA.

## Highlights

• *Paracoccus denitrificans* was studied to assess the impact of nitrite on aerobic metabolism.
• Extracellular nitrite inhibits aerobic growth of *P. denitrificans* in a pH-dependent manner.
• Increased expression of a flavohemoglobin was observed in response to extracellular nitrite.
• Analysis of a mutant established flavohemoglobin to be involved in nitrite tolerance.
• Only ∼ 1.5% of the genome was differentially regulated in response to nitrite ‘stress’.

## Graphical abstract

**Figure:**
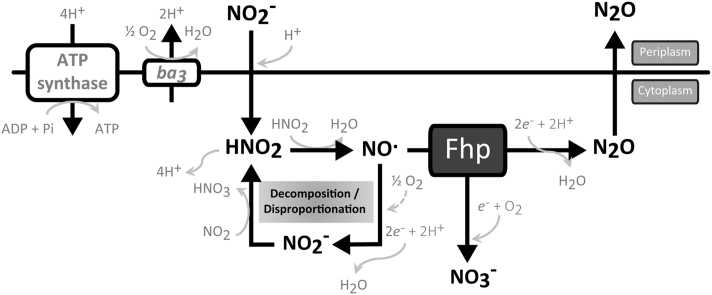

## 1 Introduction

The biocidal effect of nitrite in equilibrium with free nitrous acid (nitrite/FNA) has recently been harnessed in wastewater treatment to control unwanted growth of microorganism communities (Vadivelu et al., 2007, Wang et al., 2016). However, accumulation of nitrite can inhibit the metabolism of several groups of bacteria involved in nitrogen removal in wastewater treatment plants, including ammonia oxidisers (NH_3_ → NO_2_^−^) and denitrifiers (NO_2_^−^ → N_2_) that together can remove harmful levels of reactive nitrogen species from wastewater effluents (Almeida et al., 1995a, Anthonisen et al., 1976, Vadivelu et al., 2006a, Vadivelu et al., 2006b) and can impact on polyphosphate accumulators (Fux et al., 2003, Zhou et al., 2007, Zhou et al., 2008). Nitrite inhibition may be attributable to the protonated conjugate acid of nitrite, free nitrous acid (FNA; p*K*_a_ = 3.25), which can cross the cytoplasmic membrane as the freely diffusing uncharged lipophilic species. Once it is in the cytoplasm FNA can disproportionate to form cytotoxic reactive nitrogen species such as nitric oxide (NO) and, if oxygen is present, peroxynitrite. Extensive work by Ye et al. (2010) has suggested that this is likely to be the case for many observations of nitrite linked growth inhibition, with both catabolic and anabolic processes being affected. For example, in a mixed culture of enriched polyphosphate accumulating and glycogen accumulating bacteria, comprising largely of *Competibacter*, consumption of polyhydroxyalkanoate and glycogen production were both impacted, with complete inhibition of growth occurring at an FNA concentration of 0.14 μM (Ye et al., 2010). In a study with *Accumulibacter* 0.42 μM FNA completely inhibited phosphate uptake (Zhou et al., 2010). This was corroborated by Jiang et al. (2011) who saw a 75% decrease of biofilm after exposure to 0.42–0.61 μM FNA.

Denitrifying bacteria reduce nitrate sequentially via nitrite, NO and nitrous oxide to nitrogen. These reductive reactions are an alternative to oxygen respiration and are coupled to the generation of a proton motive force and so to cell maintenance and growth under anoxic conditions (Berks et al., 1995, Richardson, 2000). The generation of reactive nitrogen species is an ‘occupational hazard’ for denitrifying bacteria since both nitrite and nitric oxide are cytotoxins. Under anoxic conditions denitrifying bacteria express respiratory enzymes that can serve to reductively destroy extra-cytoplasmic nitrite and NO that are generated in the periplasm from nitrate reduction or produced in a microbial community by other organisms. These are the nitrite reductase (Nir) and the nitric oxide reductase (Nor). Indeed, in *P. denitrificans nir* and *nor* gene expression is co-regulated by the same transcriptional regulator, NnrR, an NO sensor (Van Spanning et al., 1995). This ensures that the production and consumption of reactive nitrogen species is tightly coupled. However, expression of the *nir* and *nos* systems is repressed by oxygen and activity of the enzymes themselves is inhibited by oxygen. Denitrifying bacteria live at the oxic-anoxic interface in many environments and the nitrite and nitrate that they use as substrates for denitrification arise from the aerobic nitrification process. Thus denitrifying bacteria will frequently be exposed to nitrite/FNA in oxic environments leading to the generation of additional reactive nitrogen species as a consequence. With this in mind we have sought to explore the effect of nitrite/FNA on aerobic *P. denitrificans* metabolism and we report here the identification of a cytoplasmic system that contributes to survival at high nitrite/FNA concentrations similar to those reached in some wastewater treatment processes. The research provides molecular information on the response of a denitrifying organism to nitrite/FNA that can inform those in the water industry assessing the biological impact of nitrite/FNA in various applications.

## 2 Materials and methods

### 2.1 Bacterial strains, media and plasmids

*P. denitrificans* PD1222, derivative strains and *Escherichia coli* were cultured using Lysogeny broth (LB) media containing rifampicin (25 μg mL^−1^), kanamycin (25 μg mL^−1^) or gentamicin (25 μg mL^−1^), where appropriate. For growth experiments, a minimal medium was used as described previously (Felgate et al., 2012) with varying levels of nitrate and nitrite and 30 mM succinate and 10 mM NH_4_Cl for carbon and nitrogen sources for growth, respectively. Continuous culture experiments were performed as described by Felgate et al. (2012) Aeration was maintained throughout to maintain a concentration of 0.236 mM (% air saturation).

### 2.2 Aerobic batch culture techniques

Aerobic growth profiles were measured in a 96 well plates format (FLUOstar Omega, UK) containing 100 μL minimal medium and 1% inoculum. Plates were incubated at 30 °C with orbital shaking at 400 rpm. Growth was monitored every 0.5 h as optical density (OD) at 600 nm and adjusted to a pathlength of 1 cm. Additional aerobic growth profiles were performed in shaking flasks to facilitate liquid, gas and RNA sampling based on using 50 mL of minimal medium added into a 250 mL conical flask and incubated at 30 °C with orbital shaking (200 rpm). Each flask was sealed using a gas permeable foam bung and aluminium foil lightly pressed around the edge to enable gas exchange. Bacterial growth was monitored spectrophotometrically using an Eppendorf^®^ Biophotometer at 600 nm. Growth rates and profiles, here termed as apparent value of exponential growth rate μ_app_, were calculated based on a semi-log plot of OD_600nm_ measurements as a function of time, using the OriginPro 9.0 (OriginLab). The Y_max_ is defined as the maximum OD _600nm_ reached on the growth curve. All growth curves presented are derived from an average of 6 independent experiments and error bars are ± the standard error.

### 2.3 Aerobic continuous culture technique

Continuous cultures were established in 2.5 L bio-reactors (BioFlo 310, New Brunswick Scientific) similarly to the study of Felgate et al. (2012). Bacteria were incubated in 1.5 L minimal media saturated with air. Vigorous agitation (400 rpm) and continuous air flow maintained the dissolved oxygen levels at 100% (air saturation). Temperature and pH were maintained at 30 °C and 7.5, respectively, through-out the incubation. A typical continuous culture run consists of an initial batch phase for 22 h followed by continuous culture with a dilution rate set at 0.05 h^−1^.

### 2.4 Analytical methods

Concentrations of extracellular nitrate and nitrite were determined with high performance liquid chromatography (HPLC). The Dionex^®^ ICS-900 HPLC system was fitted with a 2 mm × 250 mm IonPac^®^ AS22 column and a DS5 conductivity sensor. The system was equilibrated with 4.5 mM sodium carbonate (Na_2_CO_3_) and 1.4 mM sodium bicarbonate (NaHCO_3_). The regenerant used was 10 mM sulphuric acid (H_2_SO_4_). Calculated based on the p*K*_a_ (3.25) of the equilibrium for NO_2_^−^/FNA using a formula derived from the Henderson-Hasselbalch equation. Nitrous oxide detection was carried out using a Perkin Elmer Clarus^®^ 500 gas chromatographer equipped with an electron capture detector (ECD) and Elite-PLOT Q using nitrogen as the carrier gas: nitrogen and a mixture of 95% argon/5% methane as the make-up gas as in Sullivan et al. (2013). Calibration gases were acquired from Scientific and Technical Gases Ltd, UK.

### 2.5 Construction of *fhp*^−^ deficient *P. denitrificans*

An in-frame deletion of *pden*_1689 (*fhp*) was generated using the mobilisable suicide plasmid pK18*mobsacB* by allelic exchange via homologous recombination, essentially as described in Sullivan et al. (2013). Briefly, regions directly upstream and downstream of the DNA to be deleted were amplified by PCR using oligonucleotides incorporating restriction enzyme sites (Supplementary Table 2). These were cloned into pK18*mobsacB* and the resultant plasmid pKH001 was conjugated into *P. denitrificans* PD1222 via triparental mating with *E. coli* harbouring the plasmid pRK2013 Transconjugants were selected first by kanamycin resistance, and subsequently, by selection on LB media containing sucrose. Double-cross over events were screened by PCR and isogenic *fhp*^*-*^ *P. denitrificans* was verified by PCR and sequencing.

### 2.6 Complementation of *fhp in trans*

To complement the *fhp* mutant *P. denitrificans* strain *in trans*, oligonucleotides were used to amplify the entire *fhp* locus plus 290 bp of DNA upstream of the ATG start codon to include any native *cis-*acting elements required for expression. The PCR product was cloned into the MCS of the broad host-range plasmid *p*OT2 (Allaway et al., 2001) and conjugated into *fhp*^−^ *P. denitrificans* as described above. Prior to growth experiments, persistence of the *fhp*:pOT2 construct was maintained by selecting on gentamicin (20 μg mL^−1^).

### 2.7 RNA extraction from *P. denitrificans*

RNA was isolated from biological triplicates at similar growth phases for each comparison. Surfaces and equipment used were treated for RNase contamination using RNaseZAP^®^ (Ambion) or autoclaved. For harvesting RNA, cultures of bacteria were stabilised by the addition of 0.4 volumes of phenol–ethanol (5%:95%, respectively) and incubated for 1 h on ice. RNA extraction and purification was carried out using the SV Total RNA Isolation System from Promega^®^ Z3100 as per manufacturer's instructions for both qRT-PCR and microarray analysis. Quantification of RNA yield was obtained spectrophotometrically in a Thermo Scientific NanoDrop 2000™ Spectrophotometer at 260 nm. DNA contamination was removed using Ambion™ TURBO™ DNase. RNA integrity and degradation was checked by electrophoresis using the Experion^(TM)^ automated electrophoresis system and Prokaryotic StdSens RNA analysis kit (*BioRad*), and DNA contamination of RNA samples was analysed by PCR using RNA isolations as template and Bioline MyTaq Polymerase. The absence of PCR products confirmed the absence of DNA in RNA samples, compared to positive and negative controls.

### 2.8 Quantitative real-time reverse transcription PCR

Quantitative real-time reverse transcription PCR (qRT-PCR) was used to quantify mRNA for gene expression profiling and validation of type II microarray analysis. Total RNA was reverse transcribed using Superscript III^TM^ (Invitrogen, UK) according to the manufacturer's protocol. Oligonucleotides for the target genes (Supplementary Table 1) were selected based on the genome of *P. denitrificans* PD1222 using Primer3 and ordered through Eurofins MWG^®^ Operon (DE). Gene expression was assessed with a Bio-Rad^®^C1000 Thermal Cycler and CFX96 Real-time PCR detection system using SensiFAST™ SYBR^®^ Green Master Mix (Bioline, UK) according to the manufacturer's instructions The PCR assays were subjected to melt-curve analyses to identify/eliminate non-specific PCR products. Gene expression was normalised to the glyceraldehyde 3-phosphate dehydrogenase (GAPDH) housekeeping gene using primers previously described (Sullivan et al., 2013) and relative expression ratios were calculated using primer efficiencies derived from standard curves as described previously (Pfaffl, 2001). The melt-curve analyses for GAPDH and *fhp* amplicons are presented in the supplementary data (Supplementary Fig. 1). All qRT-PCR experiments conformed to the Minimum Information of Quantitative Real-time PCR Experiment (MIQE) guidelines.

### 2.9 Microarray analyses

RNA was reverse transcribed (RT) to cDNA using Agilent Technologies AffinityScript™ and then labelled with Cy5-dCTP Red fluorophore (Amersham). Genomic DNA was extracted using the QIAamp^®^ DNA Mini Kit from QIAGEN^®^ following manufacturer's instructions. *P. denitrificans* PD1222 reference genomic DNA was labelled with Cy5-dCTP (Amersham) using the BioPrime DNA labelling system and Klenow enzyme (Life Technologies). Microarray hybridisations were carried out as essentially described previously (Sullivan et al., 2013).

Microarray slides were scanned with a GenePix 4000A scanner (Axon, USA). The fluorescence intensity was imaged with Genepix Pro 7.0 Software. Saturation tolerance was typically set at 0.05 or 5%. Wavelengths were set at 635 and 532 for green/red laser beam. Fluorescence intensity of each dot was quantified by subtraction of background fluorescence and by red/green (Cy5/Cy3) ratio. Intensity values were normalised with the Batch Anti-Banana Algorithm in R (BABAR) algorithm and software package. Statistical analysis of the microarray datasets was done with Genespring 7.3 (Agilent, UK). Genes were filtered with a ≥2 fold expression filter (p ≤ 0.1) and exported into Microsoft Excel.

## 3 Results and discussion

### 3.1 The effect of extracellular nitrite on aerobic growth of *P. denitrificans* in batch cultures

**Fig. 1:**
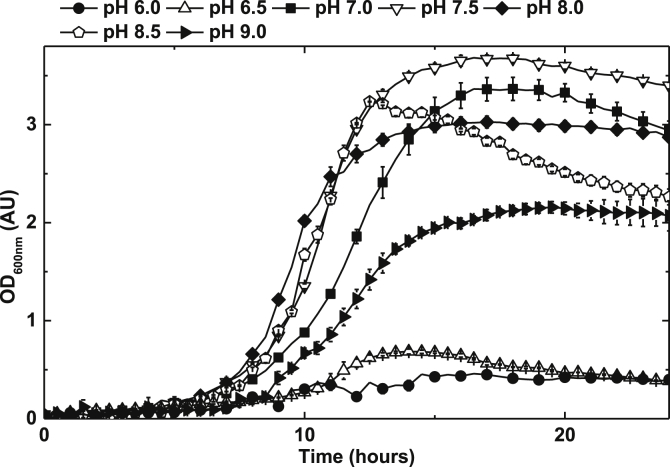
The effect of pH on the aerobic growth of *P. denitrificans* PD1222 in a defined minimal medium with pH ranging from 6.0–9.0. 100 μl cultures in 96-well plates were agitated at 400 rpm and measured by optical density at 600 nm (OD_600nm_). Bars denote standard error of at least three independent biological replicates (n = ≥3).

**Fig. 2:**
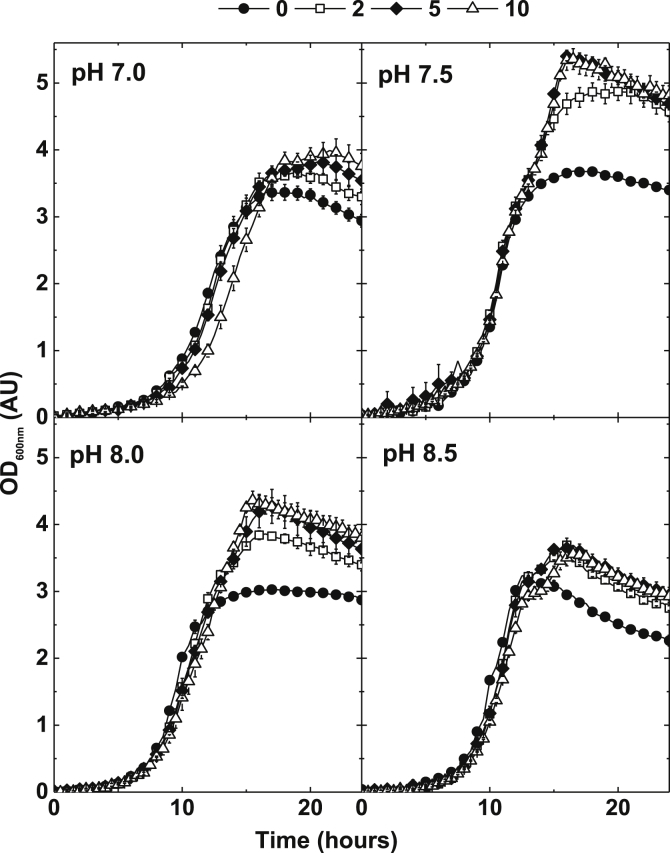
Stimulation of aerobic growth of *P. denitrificans* PD1222 with elevated concentrations of nitrite (shown in legend) in a defined minimal medium at pH of 7.0, 7.5, 8.0 and 8.5. 100 μl cultures in 96-well plates were agitated at 400 rpm and measured by optical density at 600 nm (OD_600nm_). Bars denote standard error of at least three independent biological replicates (n = ≥3).

**Fig. 3:**
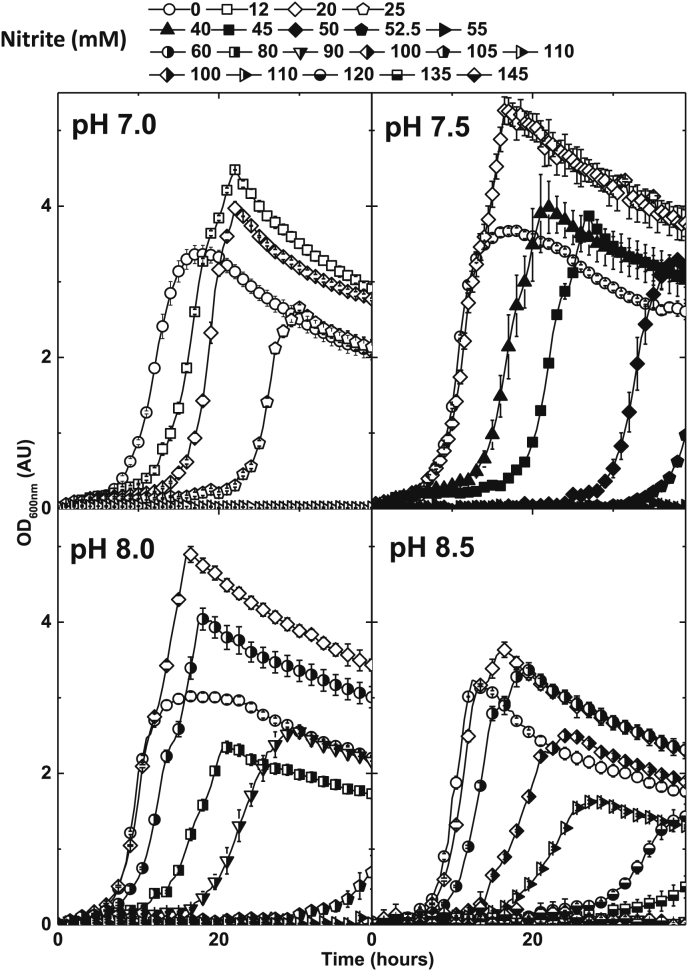
Aerobic of *P. denitrificans* PD1222 at pH 7.0, 7.5, 8.0 and 8.5, in a defined minimal medium with nitrite concentrations in the range of 0 mm to 145 mM. 100 μl cultures in 96-well plates were agitated at 400 rpm and measured by optical density at 600 nm (OD_600nm_). Bars denote standard error of at least three independent biological replicates (n = ≥3).

**Fig. 4:**
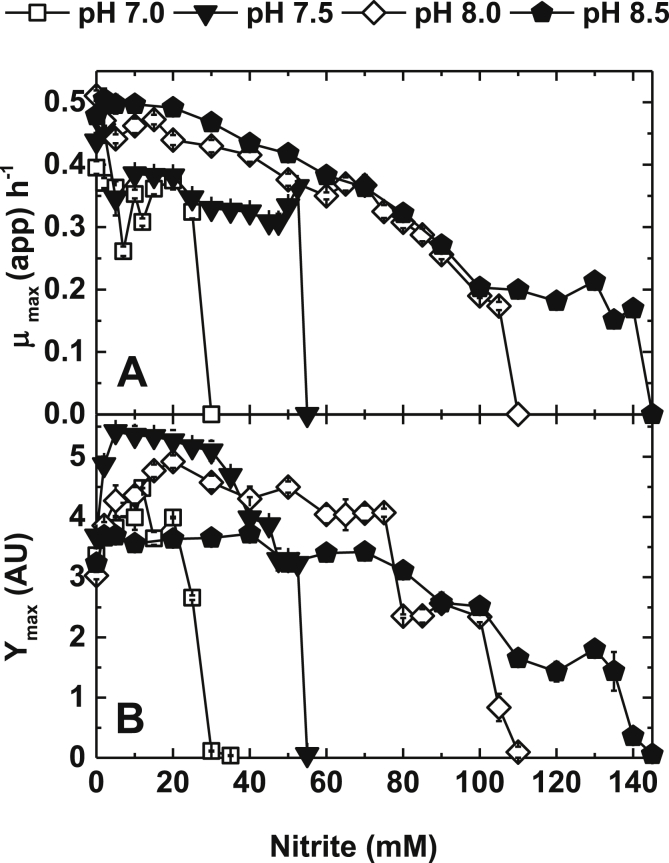
Maximum optical density produced (Y_max_ AU) from a variety of nitrite concentrations from the growth of *P. denitrificans* PD1222 at pH 7.0, 7.5, 8.0 and 8.5, in a defined minimal medium. 100 μl cultures in 96-well plates were agitated at 400 rpm and measured by optical density at 600 nm (OD_600nm_). Bars denote standard error of at least three independent biological replicates (n = ≥3).

*P. denitrificans* was grown in aerated batch cultures, in either shake flask or agitated micro-titre wells, over the initial pH range of 6–9 (Fig. 1). Growth was very poor at pHs 6 and 6.5 and moderate at pH 9. Good growth to high biomass yield was observed at pHs 7, 7.5, 8 and 8.5. These pHs were therefore selected as the culture conditions for exploring the effect of extracellular nitrite on aerobic growth. Over the range of 2–10 mM initial extracellular nitrite an apparent stimulation in the growth of the cultures was observed (Fig. 2). This is a counter intuitive observation suggesting the possibility that, between 2 and 10 mM nitrite, there is an energetic gain to the cells. This gain reaches a point at which tolerance is no longer possible and the cells begin to suffer an inhibitory effect to an extent that growth is inhibited with respect to nitrite concentration addition. Over the range of 10–145 mM extracellular nitrite a degree of growth inhibition was observed that was notably pH dependent, with cultures initiated at pH 8.5 being much less sensitive to nitrite than cultures initiated at pH 7. For example, no growth was observed at 30 mM nitrite at pH 7, whilst at pH 8.5 addition of 145 mM nitrite was required to completely inhibit growth (Fig. 3, Fig. 4). Growth of *P. denitrificans* was not recovered above these pH-specific, growth-inhibiting concentrations of nitrite, therefore these data are excluded from figures for clarity. The pH dependence of sensitivity points towards FNA being a key inhibitory factor.

### 3.2 Physiological analysis of the *fhp* locus

**Fig. 5:**
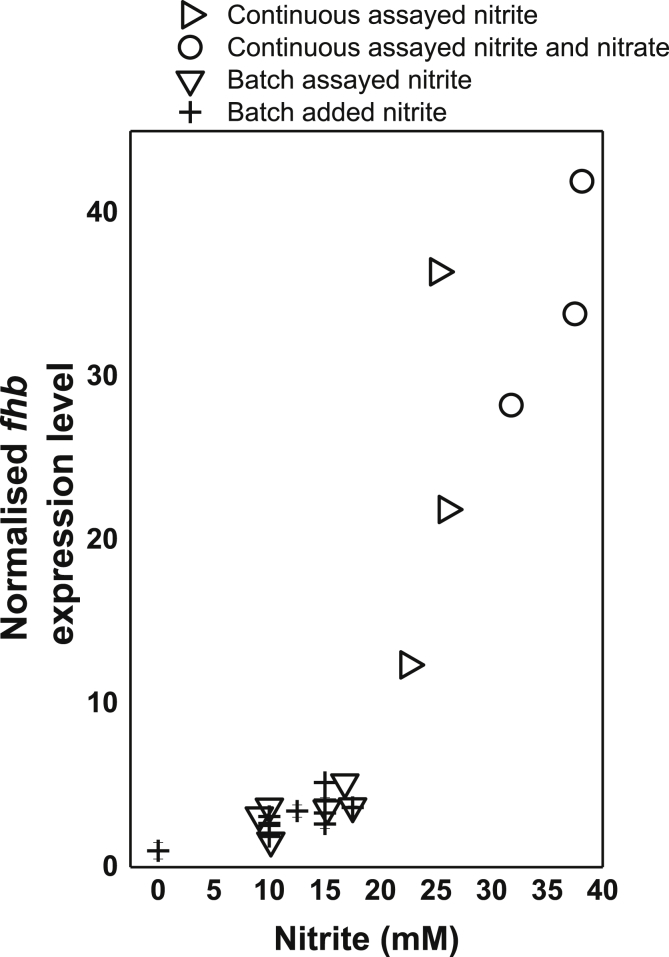
Normalised expression of the flavohemoglobin (*fhp*) (gene Pden*_*1689) in *Paracoccus denitrificans* in the presence of nitrite in batch (diamond and cross points) and continuous (triangle and circle points) cultures. Expression shows *fhp* expression in conditions of nitrite and nitrite and nitrate exposure. Expression was normalised to the glyceraldehyde 3-phosphate dehydrogenase (GAPDH) housekeeping gene. Error bars for cross points only denote standard error n = 3. ‘Assayed’ denotes nitrite (and nitrate for circle points) levels were quantified by HPLC; ‘added’ nitrite denotes the nitrite concentration added at the start of the batch culture without quantification by HPLC during the culture experiment.

Bioinformatic analysis of the *P. denitrificans* genome enabled identification of a gene (*pden_1689*) coding for a putative NO oxygenase/reductase member of the flavohemoglobin protein family epitomised by well characterised *E. coli* and *Salmonella enterica* serovar Typhimurium flavohemoglobin proteins (*pden_1689* will therefore henceforth be referred to as *fhp*). Flavohemoglobins are able to convert NO to either nitrate or nitrous oxide, depending on the presence or absence of oxygen (Poole and Hughes, 2000, Stevanin et al., 2002). The *P. denitrificans fhp* gene adjacent to a gene (divergently transcribed) that encoded for a putative homologue of the transcriptional repressor NsrR from *E. coli* which responds to nitrosative stress. Expression of *P. denitrificans fhp* was examined at pH 7.5 at a range of sub-lethal extracellular nitrite concentrations. Analysis by qRT PCR confirmed the nitrite-dependent expression of *fhp*, with expression increasing to a maximum at around 10 mM extracellular nitrite (∼2 μM FNA) (Fig. 5).

**Fig. 6:**
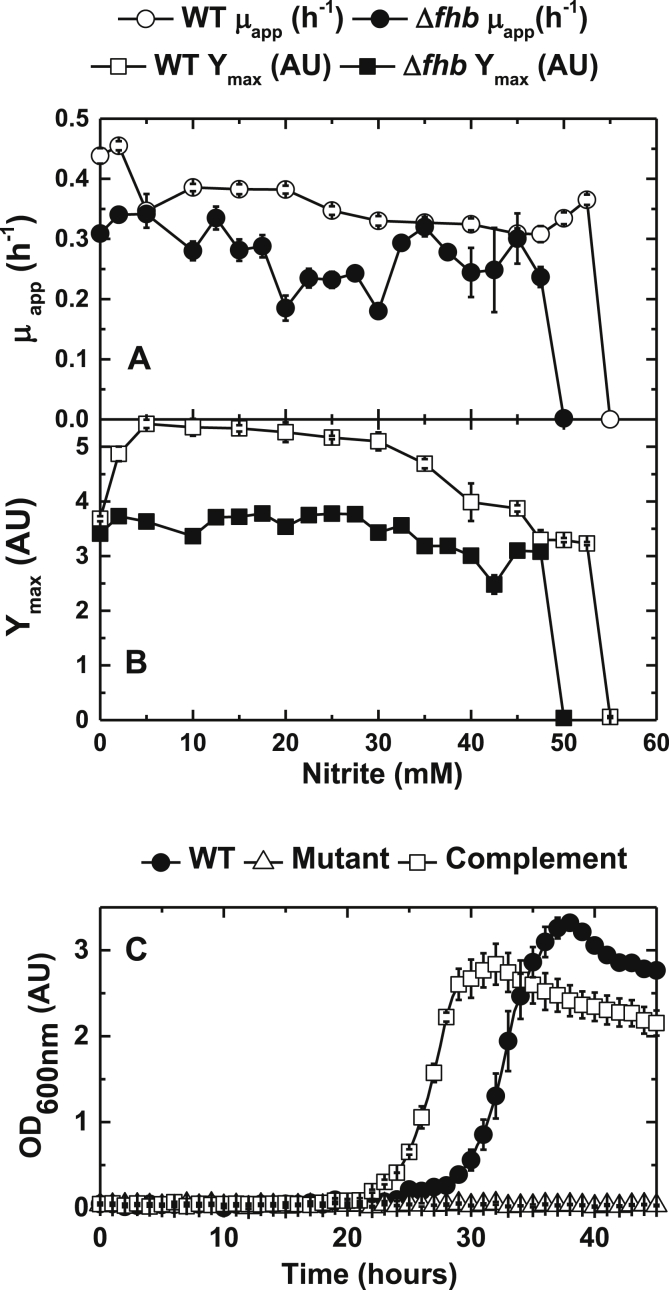
Summary of apparent maximum growth rate (A) and biomass (B) produced at various nitrite concentrations for the growth of *P. denitrificans* PD1222 and the *fhp* mutant. Growth in minimal salts media, pH 7.5, supplemented with ammonium chloride, 10 mM, sodium succinate, 30 mM and Vishniac trace element solution. Measured by optical density at 600 nm (OD_600nm_) adjusted to a pathlength of 1 cm against time (h). Error bars denote standard error (n = 3<). (C). Growth of *Paracoccus denitrificans* PD1222 wild type (WT), FHP deletion mutant and FHP complement strain in minimal salts media, pH 7.5, supplemented with 50 mM sodium nitrite. Cells were grown in 100 μl total volume with shaking at 400 RPM. OD_600nm_ was measured every 0.5 h, 1 h values shown. Error bars denote standard error n = 3.

To establish the physiological importance of *fhp* during aerobic metabolism in the presence of nitrite a deletion mutant was constructed. This mutant was able to grow at up to 45 mM nitrite, but at a consistently lower apparent growth than the WT (wild type) parent strain (Fig. 6A and B). However, unlike the parent strain the *fhp* strain was unable to grow in the presence of 50 mM nitrite (∼10 μM FNA). This capacity was recovered in full when the strain was complemented with the *fhp* gene *in trans*. (Fig. 6C).

### 3.3 Aerated continuous culture studies of *P. denitrificans* in the presence of extracellular nitrate or nitrite

**Fig. 7:**
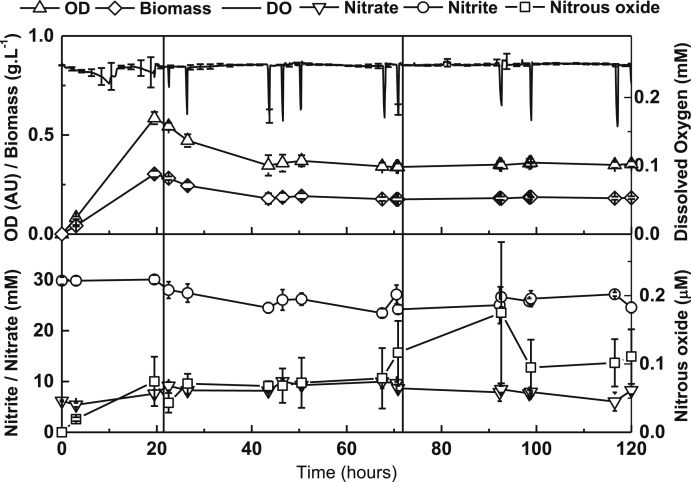
Chemostat cultures of *P. denitrificans* cultured aerobically in the presence of nitrate. Continuous culture of *Paracoccus denitrificans* PD1222 under nitrosative stress with nitrite addition to minimal salts media at pH 7.5 and 10 mM ammonium chloride (n = 3). At 22 h feed was applied at a dilution rate of 0.05 h^−1^ (vertical line at 22 h). Feed was replenished at 72 h (vertical line at 72 h). Top panel: average dissolved oxygen (DO), optical density (OD_600nm_) and biomass. Lower panel: concentration of nitrate, nitrite and nitrous oxide throughout the incubation period. (Error bars denote standard deviation; n = 3).

During shake flask or microtitre batch cultures of *P. denitrificans* it is not possible to continuously monitor or control the combination of pH, oxygen, nitrite or biomass levels. Hence in the description of the batch culture studies the external environment and the biomass levels will be changing as a function of time. To explore the effect of nitrite on steady-state *P. denitrificans* cultures, where the external pH, nitrite and biomass level can be clearly defined, we performed some additional experiments using continuous cultures (Fig. 7). The cultures were operated broadly as described by Felgate et al. (2012). Aeration was maintained throughout the culture and monitored continuously to ensure 100% air saturation and pH was maintained at 7.5. The cultures were operated as batch cultures for around 22 h during which time biomass increased. The cultures were then switched to continuous mode with medium flowing through the system to give a dilution rate of 0.05 h^−1^. Cultures were judged to have reached a biomass steady state at around 80 h (∼4 vessel volumes). Two comparisons were made: one culture was run with nitrate, which is not considered a reactive N-oxyanion and does not directly yield FNA, and the other with nitrite, which does generate FNA. A flow concentration of nitrite of 35 mM was chosen as this was just below the threshold of tolerance determined in batch cultures (Fig. 2), but would be expected to yield a nitrite/FNA stress response, as indicated by the qRT-PCR data on *fhp* expression in batch cultures (Fig. 5).

Both the aerated nitrate and nitrite cultures achieved similar biomass steady states (∼0.35 OD units) and in both cases the steady state levels of nitrate or nitrite were similar to the levels in the reservoir medium suggesting there is little, if any, aerobic denitrification occurring (Fig. 7). Thus both cultures were behaving similarly in the continuous culture systems. this was also observed with respect to expression of the genes encoding the denitrification enzymes *nirS*, *norB* and *nosZ* assessed, by qRT-PCR, which showed similar low levels in both cultures when measured at 120 h in the biomass steady-state. Again, this was consistent with the absence of ‘aerobic denitrification’. The similar behaviour of the two cultures deviated though when expression of *fhp* was assessed and which was 35-fold higher in the steady-state ‘nitrite’ compared to the ‘nitrate’ continuous cultures (Fig. 5). This confirmed that *fhp* was being expressed in order to detoxify nitrite, or a nitrite product, in the system. Since the concentration of extracellular nitrite remained constant at around 30 mM throughout the ∼100 h steady-state of the experiment it is clear that tolerance through extensive consumption of extracellular nitrite is not taking place. However, if the reactive species is actually FNA then 35 mM extracellular nitrite will equate to only around 3 μM FNA, which could diffuse into the cell and be oxidatively detoxified to nitrate by the Fhp. The 3 μM nitrate generated by this oxidation would not be detectable against the background level of nitrate in the chemostat system (Fig. 7). It was notable though that nitrous oxide, which is a potential reductive product of FNA detoxification, did accumulate to up to 0.5 μM in the steady state. Thus the up-regulation of *fhp* and the absence of significant levels of nitrite consumption suggest the intracellular FNA, rather than extracellular nitrite, is the reactive species being detoxified in the system. However, because it is not possible to observe the direct effects of nitrite and FNA in isolation due to their conjugate acid-base relationship, a contributing nitrite-specific, inhibitory effect cannot be disregarded. To test the relationship of *fhp* expression and extracellular nitrite further we set up a number of continuous culture systems at pH 7.5 with the aim of achieving steady states at a range of nitrite concentrations between in 10–35 mM. Expression of *fhp* was proportional to the steady-state level of extracellular nitrite over this range (Fig. 5).

### 3.4 The transcriptional response of oxic batch cultures of *P. denitrificans* to sub-lethal extracellular nitrite

The phenotype of the *fhp* mutant suggests a role for the flavohemoglobin in tolerance to extracellular nitrite and the resultant intracellular FNA. However, since the mutant was still able to grow at similar growth rates to WT at initial extracellular nitrite concentrations of up to 40 mM (Fig. 6A & B), there may be other systems expressed that also contribute to nitrite/FNA tolerance. To explore this further, *P. denitrificans* was cultured aerobically in the presence of sub-inhibitory concentrations of nitrite/FNA and the transcriptome established. A sub-inhibitory concentration of 12.5 mM nitrite was selected because this concentration did not affect the Y_max_ or μ_app_ of WT or *fhp* mutant cultures, but did induce *fhp* as indicated by qRT-PCR (Fig. 5). It was therefore anticipated that metabolism would have adjusted to confer resistance to the nitrite, but that the transcriptomic analysis would not be excessively complicated by cellular responses to low growth rates or cell damage that could occur at much higher, more inhibitory, nitrite concentrations. Consistent with this view, only a small number of genes (∼1.5% of the genome) were up-regulated (22 genes out of ∼5000) or down-regulated (62 genes) more than 2-fold (≥95% significance) in the cultures growing in the presence of nitrite compared to its absence. Verification of the microarray was carried out by qRT-PCR on a number of selected target genes (Table 1 & Supplementary Table 2). Consistent with the qRT-PCR data reported in the previous section, the *fhpA* gene was significantly up-regulated in the micro-array experiments. In addition, the adjacent gene *pden_1690*, divergently transcribed from *fhp*, was also up-regulated ∼2-fold in the presence of nitrite. This gene encodes for a putative transcription factor homologous to members of the NsrR family of transcriptional repressors that regulate gene expression in response to nitrosative stress. In *P. denitrificans* it seems likely that the NsrR homolog is regulating transcription of the *fhp* flavohemoglobin gene. In *E. coli* and *Salmonella* NsrR binds to the DNA upstream of the genes it regulates and prevents its transcription. It features a nitric oxide-sensitive iron-sulphur cluster, the destruction of which, by reactive nitrogen species, leads to release of NsrR protein from the DNA binding site allowing transcription to occur (Bodenmiller and Spiro, 2006).

Although widely considered as a respiratory process associated with anaerobic metabolism, *P. denitrificans* is also reported to catalyse aerobic denitrification. Since nitrite is a substrate for denitrification the expression of the genes associated with denitrification was also assessed during aerobic growth in the absence and presence of nitrite. No significant change was observed in expression of either of the two respiratory nitrate reductases (*napA* and *narG*), the respiratory nitrite reductase (*nirS*), the nitric oxide reductase (*norB*) or the nitrous oxide reductase (*nosZ*) when examined by both microarrays and qRT-PCR. This suggests that respiratory-based detoxification of nitrite during aerobic metabolism is not occurring. In addition to NirS, *P. denitrificans* also has a second nitrite induced nitrite reductase (NasB) in the cytoplasm associated with nitrite assimilation when ammonium is absent. This system was also not up-regulated by nitrite in the experiments reported here, consistent with the presence of ammonium in the growth medium and so again is not likely to be a major contributor to nitrite tolerance.

A gene encoding a respiratory protein that was affected by the presence of nitrite in the growth medium is that encoding the cytochrome *ba*_3_ oxidase (*pden_5108*), which was up-regulated by ∼2.5 fold, a change that was verified by qRT-PCR (Table 1 & Supplementary Table 2). The cytochrome *ba*_3_ oxidase mediates electron transfer between ubiquinol and oxygen. The enzyme is a membrane bound proton pump that moves protons from inside to outside the cytoplasmic membrane. One reason for up-regulation of cytochrome *ba*_3_ may therefore be a response to the uncoupling effect of FNA diffusion into the cell that effectively serves to move a proton from the periplasm into the cytoplasm and which could be an important means of tolerating lower levels of intracellular FNA. We note that the *aa*_3_-type cytochrome *c* oxidase pathway was not observed to be up-regulated in the microarray dataset, though it could also drive such proton translocation. Of the other genes that are up or down regulated it is not clear that any would be specifically involved in tolerance, with many being annotated as hypothetical proteins and a number being involved in various apparently un-related metabolic pathways. Perhaps of some note is the 2.3-fold up-regulation of *pden_4586* that is predicted to encode the chaperonin cpn10 (groES) and the 3-fold up-regulation of *pden_3605* which is predicted to code for a postulated 93 aa protein showing weak similarity (∼50%) with members of YvrJ protein family of characteristically short proteins that are part of the acid stress response in *Bacillus* species through regulation of oxalate decarboxylase acting to rebalance excess protons by catalysing the conversion of oxalate into formate and carbon dioxide with the consumption of a proton (MacLellan et al., 2009).

## 4 Conclusions

**Fig. 8:**
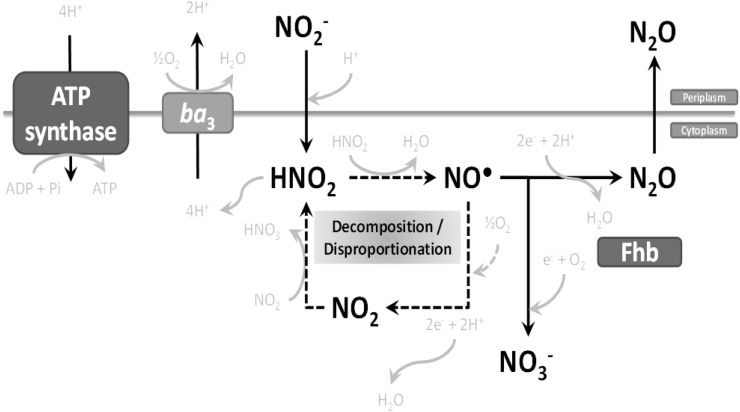
A scheme for nitrite and FNA tolerance in aerobic cultures of *P. denitrificans*.

The tolerance of a denitrifying bacterium, *P*. *denitrificans*, to extracellular nitrite during aerobic metabolism has been assessed. Nitrite exists in equilibrium with FNA and the mechanisms by which nitrite and FNA have been reported to act as cytotoxins include the following: (i) FNA can lead to the formation of reactive nitrogen and oxygen species in the cytoplasm including nitric oxide (NO), nitrogen dioxide (NO_2_), peroxynitrite (ONOO^−^), hydroxide ion (OH^−^) and hydrogen peroxide (H_2_O_2_), all of which exhibit toxicity towards bacterial cells, damaging cellular function and metal centres in protein active sites, disrupting biofilm attachment and causing cell death; (ii) FNA has been suggested to act as an uncoupler whereby it acts to circumvent the ATP synthesis as a result of a short-circuit formed by FNA transporting protons across the inner membrane and back into the cell and so increasing the conductance of the cytoplasmic membrane; and (iii) FNA may be able to directly inhibit electron carriers (Almeida et al., 1995a, Almeida et al., 1995b, Barraud et al., 2006). These possible cytotoxic processes are shown in the schematic (Fig. 8). In the present work the increased sensitivity of aerobic metabolism of *P. denitrificans* to nitrite at pH 7 compared to pH 8.5 is consistent with FNA being the active component and sensitivity was in the range of 1–6 μM FNA depending on the growth condition. In principle *P. denitrificans* has enzymes in the periplasm that are able to contribute to detoxification of nitrite and nitric oxide, namely the nitrite reductase (NirS) and NO reductase (NorB). The genes encoding these systems were not induced under oxic conditions in the presence of nitrite and nitrite levels remained high throughout growth of cultures indicating that it itself was not the toxic species and was tolerated at levels up to 140 mM at high pH. Rather, the key response to extracellular nitrite was induction of a gene predicted to encode a flavohemoglobin, which could potentially oxidise nitric oxide to nitrate or reduce nitric oxide to nitrous oxide. This gene was co-located with a putative NO-responsive regulator NsrR that is known to regulate a number of NO-detoxification systems in *E. coli* and *Salmonella* (Spiro, 2007). The active range of nitrite/FNA observed in some complex community environments is similar to that identified here in the pure cultures of *P. denitrificans.* It will be of interest to explore gene expression in complex communities in wastewater-treatment systems exposed to sub-lethal FNA concentrations to examine if there is evidence for tolerance to FNA associated with increased expression of flavohemoglobins to further correlate the studies.

## Figures and Tables

**Table 1:** Verification of the microarray analysis by qRT-PCR transcriptional analysis of selected genes of *P. denitrificans* grown aerobically at pH 7.5 in the presence of 12.5 mM nitrite.

Gene identifier (Pden_)	Gene name	Microarray transcription	qRT-PCR transcription^a^	±SD
3605	Acid stress	3.00	2.06	1.27
1690	BadM reg.	2.16	3.61	1.88
1129		0.21	0.43	0.25
5108	Cyt ba3	2.45	2.68	0.89
1629	sigma-24	0.45	0.39	0.13
3429	dctM	0.44	0.47	0.10
1689	fhp	2.17	3.43	0.37
4721	napA	0.89	0.38	0.10
4236	narG	1.72	0.84	0.15
2487	nirS	0.59	1.12	0.13
2483	norB	0.54	0.65	0.05
4452	nasB	0.93	1.18	0.14
4465	gapdh^a^	1.18	0.98	0.04
0342	polB	1.12	1.03	0.04

±SD standard deviation n = 3 biologically independent replicates (additional 3 technical replicates).

a All qRT-PCR results use gapdh as housekeeping gene for normalisation, with the exception of gapdh itself whose transcription was verified with polB as housekeeping gene.
