# Supplementary material for: The metabolic impact of extracellular nitrite on aerobic metabolism of Paracoccus denitrificans
Source: Water Res. 2017 Apr 15;113:207–14. doi: 10.1016/j.watres.2017.02.011 (PMC5339346; doi:10.1016/j.watres.2017.02.011)
Supplement: Supplementary file 1 [file mmc1.docx]

**Manuscript: WR37267.**

**Title: The metabolic impact of extracellular nitrite on aerobic metabolism of Paracoccus denitrificans.**

**Authors: David Richardson; Kathryn Hartop; Matthew Sullivan; Georgios Giannopolous; Andrew Gates; Phillip Bond; Ziguo Yuan; Thomas Clarke, Gary Rowley.**

**Corresponding author: Prof David Richardson.**

**Supplementary Table 1**

**Oligonucleotides used in gene deletion and complementation**

| **Name** | **Sequence** | **Use** |
| --- | --- | --- |
| fbp_5fln_F2 | ag**cccggg**AGAATTACGGCTATTATCTG | 5’ flanking region *fhp-* deficient *P. denitrificans* |
| fbp_5flnR1 | ag**tctaga**CGATATCAAGACCATGAG |  |
| fbp_3fln_F1 | ag**tctaga**CCAAGCGCAACTATTCGAT | 3’ flanking region *fbp* deletion mutant |
| fbp_3fln_R1 | ag**ctgcag**CGATCAGGTAGAGCATGACC |  |
| fbp_qPCR1F | AGGTTCCGGTGCATTACATC | qRT-PCR |
| fbp_qPCR1R | GTCTGGTGGAAATCGGTGAC |  |
| fbp_qPCR2F | GGTCTTGATATCGTGCGTGA |  |
| fbp_qPCR2R | GTTTGCATAAGCCAGGATCG |  |
| fbp_compF1 | ca**tctaga**GACGACATGGACGATCCG | Insertion of *fbp* gene to *p*OT2 vector |
| fbp_compR1 | **tctaga**CATCAACGATCTGGTGAAC |  |
| fbp_checkF1 | GCATCCTGATTGCATGTTTCTG | Confirmation of *fbp* complement to *P. denitrificans* |
| fbp_checkR1 | CCATTCCTCGGTGATCAGCC |  |
| *Includes restriction sites in lower case bold. Start codon underlined.* | | |

Supplementary Table 2: Gene regulation in *P. denitrificans* PD1222 under nitrosative stress conditions normalised to standard growth conditions. Growth conditions are minimal media at pH 7.5 with 10 mM NH4Cl and 30 mM sodium succinate, plus nitrite: 12.5 mM sodium nitrite addition. Showing 84 genes A: highly transcribed in the presence of nitrite and B: highly down-regulated in the presence of nitrite. A: with 95% significance. B: 90% significance. Those highlighted in orange have been used for further bioinformatic analysis. Annotations are based on bioinformatic BLAST analysis for *Paracoccus*.* Putative protein with uncharacterised function.

| **A** | **Mean normalised transcription ^a^** | | | | | | |
| --- | --- | --- | --- | --- | --- | --- | --- |
| **Gene**  **(Pden_)** | **Minus nitrite** | **±SD** | **Plus nitrite** | **±SD** | **Fold change ^b^** | | **Gene annotation** |
| 3605 | 0.13 | 0.03 | 0.38 | 0.08 | 3.00 | putative protein possible YvrJ protein family | |
| 3764 | 0.12 | 0.03 | 0.35 | 0.13 | 2.89 | putative protein | |
| 2988 | 0.52 | 0.15 | 1.44 | 0.29 | 2.74 | putative protein | |
| 5108 | 0.98 | 0.23 | 2.40 | 0.42 | 2.45 | cytochrome ba3 quinol oxidase subunit 2 | |
| 3750 | 0.22 | 0.04 | 0.53 | 0.11 | 2.43 | putative protein | |
| 4586 | 16.33 | 1.44 | 38.20 | 0.81 | 2.34 | chaperonin Cpn10 | |
| 4576 | 0.25 | 0.05 | 0.58 | 0.04 | 2.31 | conserved putative protein | |
| 2112 | 0.08 | 0.01 | 0.19 | 0.04 | 2.21 | flagellar hook-basal body complex protein FliE | |
| 0744 | 37.08 | 6.85 | 81.66 | 7.63 | 2.20 | LSU ribosomal protein L1P | |
| 1689 | 0.45 | 0.11 | 0.97 | 0.07 | 2.17 | globin FHP | |
| 1690 | 0.59 | 0.19 | 1.27 | 0.17 | 2.16 | transcriptional regulator, BadM/Rrf2 family | |
| 4799 | 0.48 | 0.11 | 1.04 | 0.06 | 2.17 | enoyl-CoA hydratase / short chain enoyl-CoA hydratase | |
| 3118 | 1.34 | 0.14 | 2.89 | 0.32 | 2.15 | methionine aminopeptidase, type I | |
| 4589 | 0.50 | 0.04 | 1.08 | 0.27 | 2.15 | putative protein | |
| 3211 | 0.52 | 0.11 | 1.12 | 0.18 | 2.14 | aldehyde dehydrogenase | |
| 3460 | 0.76 | 0.21 | 1.60 | 0.15 | 2.12 | ABC polyamine/opine transporter, periplasmic substrate-binding | |
| 4895 | 0.21 | 0.00 | 0.45 | 0.06 | 2.12 | BFD domain protein (2Fe-2S)-binding domain protein | |
| 2787 | 3.98 | 1.13 | 8.16 | 0.25 | 2.05 | smr protein/MutS2 | |
| 4577 | 0.30 | 0.08 | 0.62 | 0.13 | 2.08 | short-chain dehydrogenase/reductase SDR | |
| 2409 | 1.15 | 0.13 | 2.39 | 0.15 | 2.07 | putative protein | |
| 3216 | 2.52 | 0.69 | 5.06 | 0.26 | 2.01 | tartronate semialdehyde reductase | |
| 4930 | 0.56 | 0.13 | 1.13 | 0.08 | 2.02 | 2-keto-3-deoxy-phosphogalactonate aldolase | |

| **B** | **Mean normalised transcription ^a^** | | | | | | |
| --- | --- | --- | --- | --- | --- | --- | --- |
| **Gene**  **(Pden_)** | **Minus nitrite** | **±SD** | **Plus nitrite** | **±SD** | **Fold change ^b^** | | **Gene annotation** |
| 1707 | 2.86 | 1.66 | 0.47 | 0.33 | | 0.16 | conserved hypothetical protein |
| 1483 | 0.93 | 0.49 | 0.16 | 0.02 | | 0.17 | conjugal transfer protein TrbC |
| 2366 | 7.87 | 1.09 | 1.36 | 0.07 | | 0.17 | aldehyde dehydrogenase (NAD(+)) |
| 1725 | 4.66 | 3.01 | 0.88 | 0.60 | | 0.19 | putative protein |
| 1129 | 1.69 | 0.48 | 0.35 | 0.12 | | 0.20 | von Willebrand factor, type A |
| 2368 | 7.78 | 3.11 | 1.65 | 0.10 | | 0.21 | protein of unknown function DUF779 |
| 3408 | 0.28 | 0.13 | 0.07 | 0.03 | | 0.24 | putative protein |
| 2367 | 5.02 | 1.28 | 1.24 | 0.16 | | 0.25 | alcohol dehydrogenase GroES domain protein |
| 1566 | 17.52 | 9.10 | 4.82 | 1.43 | | 0.28 | cobalamin-5'-phosphate synthase |
| 0944 | 1.55 | 0.44 | 0.49 | 0.10 | | 0.32 | transcriptional regulator, TetR family |
| 0996 | 0.32 | 0.15 | 0.10 | 0.03 | | 0.32 | gene transfer agent |
| 0020 | 1.05 | 0.19 | 0.34 | 0.03 | | 0.32 | pyrrolo-quinoline quinone |
| 1097 | 0.57 | 0.19 | 0.19 | 0.08 | | 0.33 | conserved putative protein |
| 0263 | 0.87 | 0.40 | 0.29 | 0.05 | | 0.34 | DNA-cytosine methyltransferase |
| 2445 | 0.73 | 0.22 | 0.26 | 0.04 | | 0.35 | protein of unknown function DUF796 |
| 0260 | 0.54 | 0.18 | 0.19 | 0.07 | | 0.36 | putative protein |
| 4290 | 1.85 | 0.62 | 0.68 | 0.14 | | 0.37 | conserved putative protein |
| 1225 | 0.62 | 0.20 | 0.23 | 0.04 | | 0.37 | putative protein |
| 1461 | 0.45 | 0.15 | 0.17 | 0.07 | | 0.38 | GCN5-related N-acetyltransferase |
| 0366 | 0.31 | 0.10 | 0.12 | 0.02 | | 0.39 | phage head-tail adaptor, putative |
| 1486 | 0.21 | 0.08 | 0.08 | 0.03 | | 0.39 | tonB-dependent receptor |
| 3263 | 0.48 | 0.18 | 0.19 | 0.06 | | 0.39 | hypothetical protein |
| 1703 | 0.80 | 0.15 | 0.32 | 0.10 | | 0.40 | quinohemoprotein amine dehydrogenase, 60 kDa subunit |
| 1537 | 0.23 | 0.07 | 0.09 | 0.03 | | 0.41 | putative protein |
| 2701 | 0.45 | 0.09 | 0.19 | 0.04 | | 0.41 | putative protein |
| 2118 | 0.73 | 0.07 | 0.31 | 0.09 | | 0.42 | conserved putative protein |
| 1210 | 0.64 | 0.18 | 0.27 | 0.05 | | 0.42 | HupE/UreJ protein |
| 2205 | 18.03 | 5.68 | 7.64 | 1.09 | | 0.42 | heat shock protein Hsp20 |
| 3010 | 1.92 | 0.72 | 0.81 | 0.32 | | 0.42 | periplasmic binding protein |
| 1477 | 0.49 | 0.18 | 0.21 | 0.07 | | 0.43 | conjugal transfer protein |
| 2467 | 0.71 | 0.22 | 0.30 | 0.07 | | 0.43 | response regulator receiver protein |
| 3069 | 0.53 | 0.09 | 0.23 | 0.01 | | 0.43 | transcriptional regulator, LysR family |
| 0025 | 1.08 | 0.40 | 0.47 | 0.13 | | 0.43 | putative protein |
| 0159 | 1.11 | 0.28 | 0.48 | 0.10 | | 0.44 | putative protein |
| 0390 | 0.74 | 0.16 | 0.32 | 0.03 | | 0.44 | protein of unknown function DUF983 |
| 3429 | 0.43 | 0.13 | 0.19 | 0.02 | | 0.44 | TRAP dicarboxylate transporter, DctM subunit |
| 1629 | 1.91 | 0.53 | 0.85 | 0.03 | | 0.45 | RNA polymerase, sigma-24 subunit, ECF subfamily |
| 0219 | 0.31 | 0.10 | 0.14 | 0.03 | | 0.45 | putative protein |
| 3530 | 1.06 | 0.21 | 0.48 | 0.07 | | 0.45 | siderophore-interacting protein |
| 2652 | 1.90 | 0.52 | 0.85 | 0.05 | | 0.45 | flagellar biosynthetic protein FliP |
| 0021 | 1.40 | 0.22 | 0.63 | 0.09 | | 0.45 | putative protein |
| 0276 | 0.57 | 0.15 | 0.26 | 0.04 | | 0.45 | conserved putative sugar-binding protein |
| 1606 | 0.26 | 0.06 | 0.12 | 0.01 | | 0.46 | methyltransferase type 12 |
| 3748 | 0.57 | 0.20 | 0.26 | 0.07 | | 0.46 | putative protein |
| 3521 | 0.51 | 0.17 | 0.24 | 0.03 | | 0.46 | tonB-dependent siderophore receptor |
| 1911 | 9.32 | 2.59 | 4.36 | 0.48 | | 0.47 | putative protein |
| 1446 | 0.37 | 0.06 | 0.17 | 0.01 | | 0.47 | major facilitator superfamily MFS_1 |
| 1126 | 0.95 | 0.24 | 0.45 | 0.13 | | 0.47 | conserved putative protein |
| 0102 | 0.21 | 0.07 | 0.10 | 0.02 | | 0.47 | phage transcriptional regulator, AlpA |
| 4193 | 0.46 | 0.11 | 0.22 | 0.05 | | 0.47 | putative protein |
| 3983 | 2.50 | 0.56 | 1.19 | 0.05 | | 0.48 | conserved putative protein |
| 2634 | 0.43 | 0.09 | 0.21 | 0.01 | | 0.48 | HNH endonuclease |
| 3464 | 1.32 | 0.11 | 0.63 | 0.08 | | 0.48 | ferredoxin |
| 0252 | 1.42 | 0.37 | 0.68 | 0.12 | | 0.48 | transposase (class I) |
| 1125 | 0.92 | 0.24 | 0.45 | 0.06 | | 0.48 | ATPase associated with various cellular activities, AAA_3 |
| 4243 | 2.82 | 0.76 | 1.36 | 0.19 | | 0.48 | gntR domain protein |
| 3531 | 0.57 | 0.00 | 0.28 | 0.05 | | 0.49 | periplasmic binding protein |
| 2886 | 1.15 | 0.33 | 0.56 | 0.08 | | 0.49 | conserved hypothetical protein |
| 1521 | 0.59 | 0.10 | 0.29 | 0.08 | | 0.49 | putative protein |
| 3827 | 1.52 | 0.45 | 0.74 | 0.19 | | 0.49 | putative protein |
| 3021 | 0.77 | 0.19 | 0.38 | 0.09 | | 0.49 | succinate dehydrogenase subunit C |
| 2648 | 0.54 | 0.03 | 0.27 | 0.03 | | 0.50 | flagellar basal body-associated protein FliL |

*^a^ Normalised to internal gDNA fluorescence, mean value of three biologically independent experiments, ±SD = standard deviation of biological replicates*

*^b^ Fold change derived by plus / minus nitrite mean transcription values*

Supplementary Figure 1


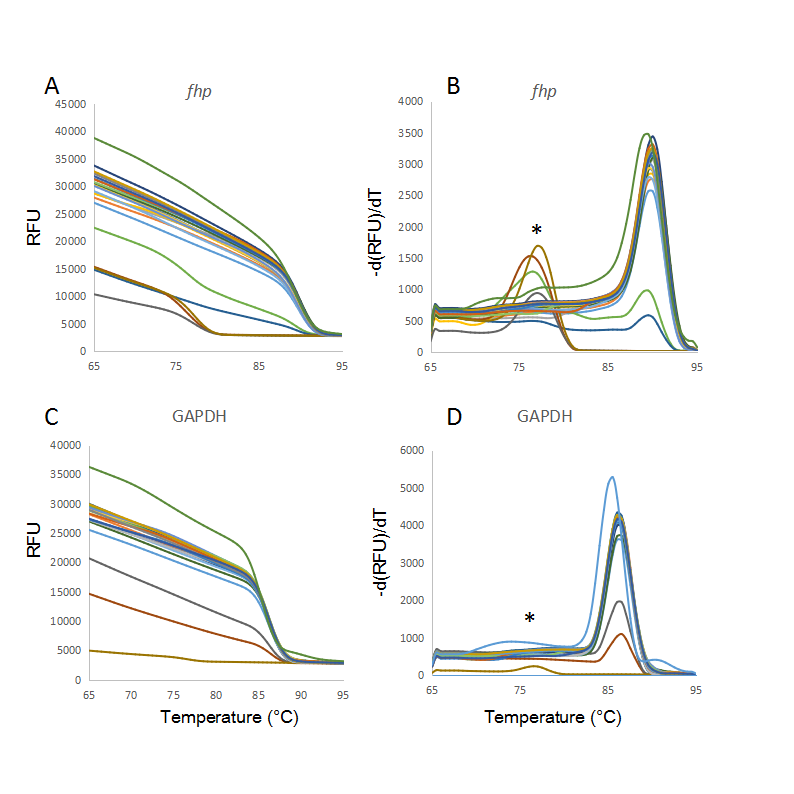


Melt Curve analysis (A and C) and melt peaks (B and D) of *fhp* and GAPDH amplicons from qRT-PCR experiments. Peaks corresponding to primer dimers (*) were confirmed by separation of PCR products via agarose gel electrophoresis. The presence of single peaks > 85 °C indicate the primers used were highly specific and the PCR reactions contained only 1 dsDNA species per primer pair used.
